# The Impact of Subclinical Thyroid Dysfunction on Cerebrovascular Pathology: A Focus on Carotid Atherosclerosis and Stroke Severity

**DOI:** 10.3390/neurolint18100196

**Published:** 2026-10-21

**Authors:** Maria-Gabriela Vladoiu, Madalina Valeanu

**Affiliations:** 1 Department of Neurology, Dr. Holhos Multidisciplinary Hospital, Calea Moților Street, No. 61, 510034 Alba Iulia, Romania; 2 Department of Nursing, “1 Decembrie 1918” University, Gabriel Bethlen Street, No. 5, 510009 Alba Iulia, Romania; 3 Department of Medical Informatics and Biostatistics, University of Medicine and Pharmacy “Iuliu Hatieganu” Cluj-Napoca, Victor Babeş Street, No. 8, 400012 Cluj-Napoca, Romania

**Keywords:** subclinical hypothyroidism, subclinical hyperthyroidism, acute ischemic stroke, carotid atherosclerosis, atrial fibrillation, thyroid-stimulating hormone

## Abstract

**Background**: Thyroid dysfunction is a relatively common condition; however, the clinical relevance and long-term effects of subclinical thyroid dysfunction on cardiovascular and cerebrovascular morbidity remain uncertain. This study evaluated the association between subclinical thyroid dysfunction and cerebrovascular disease, focusing on lipid metabolism, carotid atherosclerosis, and stroke severity. **Methods**: This study included 80 adult patients consecutively admitted with acute ischemic stroke who were newly diagnosed with subclinical thyroid dysfunction. Patients were evaluated over a three-year period (2020–2022). Clinical data, extracranial carotid Doppler ultrasonography, and laboratory parameters (including TSH, FT4, and total cholesterol) were analyzed to establish correlations with stroke characteristics. **Results**: Subclinical hypothyroidism was identified in 83.75% of patients, while subclinical hyperthyroidism was present in 16.25%. A strong positive linear correlation was observed between serum TSH and total cholesterol levels (r = 0.794, *p* < 0.001). TSH levels significantly correlated with both the severity of carotid artery stenosis and the National Institutes of Health Stroke Scale (NIHSS) score in patients with anterior circulation stroke. Subclinical hyperthyroidism was identified as a protective factor against moderate or severe carotid atheromatosis (OR = 0.211, *p* = 0.038); however, 78% of these patients presented with newly diagnosed atrial fibrillation. **Conclusions**: Subclinical thyroid dysfunction is significantly associated with cerebrovascular pathology through distinct mechanisms. Subclinical hypothyroidism contributes to atherosclerotic vascular disease and greater stroke severity, whereas subclinical hyperthyroidism predisposes predominantly to arrhythmia-mediated embolic events.

## 1. Introduction

Thyroid dysfunction is a relatively common condition; however, its long-term effects on cerebrovascular morbidity and specific stroke outcomes have not yet been fully elucidated [1,2]. Thyroid-stimulating hormone (TSH) plays a central role in regulating the synthesis and secretion of thyroid hormones through the negative feedback mechanism of the hypothalamic–pituitary–thyroid axis. Consequently, TSH serves as a key biomarker of thyroid function: elevated TSH levels are indicative of hypothyroidism, whereas suppressed TSH levels suggest hyperthyroidism [3].

Subclinical thyroid dysfunction is defined by serum TSH concentrations outside the reference range in the presence of normal free thyroxine (FT4) levels and, in the case of subclinical hyperthyroidism, normal free triiodothyronine (T3) levels [3,4]. Notably, subclinical hypothyroidism is most frequently characterized by mildly elevated TSH levels, typically remaining below 10 mU/L. While historical approaches reserved prompt hormone replacement therapy primarily for TSH levels above 10 mU/L, recent endocrinology guidelines emphasize that therapeutic intervention is also strongly recommended for patients with TSH levels below 10 mU/L when accompanied by high cardiovascular risk, age under 65, or significant dyslipidemia [5,6]. This paradigm shift highlights that even mild subclinical thyroid failure is not a benign state, as the condition is highly prevalent in the adult population, ranging from 4% to 14%, and increases with advancing age [3,7]. In contrast, subclinical hyperthyroidism is less common, with reported prevalence rates ranging from 0.7% in the general population to as high as 10% among women [4,8].

While previous research has primarily focused on the cardiac implications of overt thyroid disease, the impact of subclinical thyroid dysfunction on the cerebral vasculature and central nervous system is increasingly recognized as a critical area of neurovascular research [9]. Thyroid hormones exert a profound influence on endothelial function, lipid homeostasis, and the coagulation cascade [10]. Subclinical hypothyroidism has been heavily implicated in the accelerated progression of atherosclerosis. It promotes endothelial dysfunction, increased arterial stiffness, and a pro-thrombotic state, all of which are major pathophysiological drivers of large-vessel ischemic stroke and carotid atheromatosis [11,12]. Conversely, subclinical hyperthyroidism, even in the absence of overt systemic symptoms, can induce subtle neuro-cardiovascular interactions, such as subclinical atrial electrical remodeling, thereby elevating the risk of cardioembolic cerebrovascular events [4,13].

Despite extensive investigation into traditional stroke risk factors, the exact clinical relevance of subclinical thyroid dysfunction in the context of acute ischemic stroke remains uncertain, representing a significant and ongoing challenge in contemporary neurological research [14,15]. Therefore, this study aimed to evaluate the association between subclinical thyroid dysfunction and cerebrovascular disease, focusing on lipid metabolism, carotid atherosclerosis, and acute stroke severity.

## 2. Materials and Methods

### 2.1. Study Design

This study included 80 adult patients diagnosed with subclinical thyroid dysfunction, consecutively admitted to the Neurology Department of the Sibiu County Emergency Clinical Hospital with a diagnosis of acute ischemic stroke or transient ischemic attack (TIA). The study was conducted over a three-year period, from January 2020 to December 2022. Patient recruitment took place over 24 months, and the remaining 12 months were allocated to patient follow-up.

### 2.2. Patient Grouping and Definitions

Patients were divided into study subgroups based on thyroid function status, utilizing our institutional laboratory reference ranges of 0.4–4.0 mU/L for TSH and 0.9–1.7 ng/dL for FT4:

Subgroup 1: Patients with subclinical hyperthyroidism, defined by FT4 within the normal reference range (0.9–1.7 ng/dL) and suppressed TSH levels (between 0.01 and 0.04 mU/L).

Subgroup 2: Patients with subclinical hypothyroidism, defined by FT4 within the normal reference range (0.9–1.7 ng/dL) and elevated TSH levels (defined as strictly >4.0 mU/L and ≤10 mU/L).

### 2.3. Clinical and Paraclinical Assessment

Demographic and clinical data were collected retrospectively and at the time of study inclusion, including age, sex, and associated pathological conditions (comorbidities). Paraclinical investigations included extracranial carotid Doppler ultrasonography performed in all patients to assess carotid atherosclerosis. Based on ultrasound findings, carotid atheromatosis was classified into three categories:

- Grade 0 (Normal/Mild): Defined by confirmed normal ultrasound findings (complete absence of macroscopic atherosclerotic plaques) or the presence of only minimal intimal thickening.
- Grade 1 (Moderate): Defined by 50–69% ICA stenosis.
- Grade 2 (Severe): Defined by ICA stenosis greater than 70%.

Laboratory parameters comprised complete blood count, serum FT4 and TSH levels, and total cholesterol levels. Throughout the study period, correlations were analyzed between thyroid-specific biological markers and the characteristics of the cerebral ischemic event.

In addition to admission metrics, the functional outcome of the patients at the time of hospital discharge was systematically assessed using the Modified Rankin Scale (mRS). For clinical comparison, the discharge outcomes were dichotomized into favorable or good clinical outcome (mRS scores 0–2) and unfavorable or poor clinical outcome (mRS scores 3–6).

Stroke etiology was systematically classified according to the TOAST (Trial of Org 10172 in Acute Stroke Treatment) criteria. Based on clinical features, extracranial ultrasound, and neuroimaging assessments, ischemic events were categorized primarily into large-artery atherosclerosis, cardioembolism, and small-vessel occlusion (lacunar infarcts) [16].

### 2.4. Inclusion and Exclusion Criteria

Inclusion criteria were as follows:

- Patients without a history of chronic ischemic stroke or previously diagnosed thyroid disease at the time of study inclusion.
- Patients with acute vascular neurological pathology and symptom onset within 24 h prior to admission.
- Patients exhibiting subclinical hyperthyroidism or subclinical hypothyroidism.

Exclusion criteria included current statin therapy, known thyroid or other endocrinological disorders, and a history of chronic ischemic stroke.

### 2.5. Statistical Analysis

Statistical analysis and data management were performed using SPSS software version 25.0 (SPSS Inc., Chicago, IL, USA). The Shapiro–Wilk test was applied to assess the normality of data distribution. Continuous variables with a normal distribution were expressed as mean ± standard deviation (SD), while non-normally distributed continuous variables (e.g., NIHSS and mRS scores) were expressed as median and interquartile range (IQR). Categorical variables were reported as absolute frequencies and percentages. To maintain the integrity of the full cohort (N = 80) and mitigate the risk of exclusion bias, missing data were handled rigorously. Missing NIHSS and mRS scores for patients with clinical TIA diagnoses were recorded as 0, reflecting the absence of permanent neurological deficit. All 80 patients underwent complete extracranial carotid Doppler ultrasonography upon admission; there were no missing ultrasound data. Patients whose ultrasound reports explicitly confirmed the absence of atherosclerotic plaques were accurately classified as Grade 0. For patients with a clinical diagnosis of transient ischemic attack (TIA), NIHSS and discharge mRS scores inherently reflect the transient nature of the neurological deficit (score of 0); these cases were retained in the general cohort but are interpreted with caution regarding overall stroke severity distributions. Correlation analyses were conducted using Pearson’s linear correlation coefficient for normally distributed data and Spearman’s rank correlation coefficient for ordinal or non-normally distributed variables. To evaluate whether serum TSH levels serve as an independent predictor for the presence of moderate-to-severe carotid atherosclerosis (Grade ≥ 1), a multivariable logistic regression model was employed. A two-tailed *p*-value < 0.05 was considered statistically significant.

Given the small sample size of the subclinical hyperthyroidism subgroup (*n* = 13), non-parametric statistical tests were preferentially utilized where appropriate to ensure robust comparative analyses. Furthermore, while a logistic regression model was employed to evaluate serum TSH levels and carotid atherosclerosis, the outcomes should be interpreted with high caution due to the inherent risk of model overfitting and statistical instability associated with small sample sizes.

## 3. Results

### 3.1. Study Population Characteristics

The study included 80 patients admitted with acute vascular neurological pathology in whom previously undiagnosed subclinical thyroid dysfunction was identified. The cohort was predominantly female (83.75%), consistent with the known epidemiological distribution of thyroid disorders. Subclinical hypothyroidism represented the majority of cases (83.75%; 67 patients), while subclinical hyperthyroidism was identified in 16.25% (13 patients) (Table 1). In all cases, thyroid dysfunction was newly diagnosed at admission.

It is important to note that while the mean FT4 concentrations differed significantly between the subclinical hyperthyroidism and subclinical hypothyroidism groups (*p* = 0.003), the values for both cohorts remained strictly within the normal physiological reference range. This statistical difference reflects the divergent physiological set-points characteristic of these distinct subclinical conditions, rather than indicating overt hormonal excess or deficiency.

From a neurological standpoint, anterior circulation ischemic stroke was the most frequent anatomical diagnosis, accounting for 45% of cases. When classified etiologically using the TOAST criteria, large-artery atherosclerosis was the predominant mechanism among patients with subclinical hypothyroidism. In contrast, cardioembolism was the defining etiology in the subclinical hyperthyroidism subgroup, driven by the high incidence of associated atrial fibrillation. Small-vessel occlusion (lacunar infarctions) represented 11% of events overall, while the remainder consisted of posterior circulation strokes and transient ischemic attacks (TIA). This distribution confirms a clear etiological divergence based on thyroid status (Table 2).

### 3.2. Thyroid Function and Lipid Profile

Serum TSH levels were abnormal in all patients, while FT4 concentrations remained within reference limits, consistent with subclinical thyroid dysfunction. Total cholesterol levels demonstrated substantial variability, ranging from 117 mg/dL to 589 mg/dL, reflecting considerable metabolic heterogeneity.

A strong positive linear correlation was observed between serum TSH levels and total cholesterol concentrations, which was statistically significant (r = 0.794, *p* < 0.001). Increasing TSH values were associated with progressively higher cholesterol concentrations, indicating a robust metabolic link between thyroid function and lipid homeostasis. Notably, the vast majority of patients (91%) exhibited hypercholesterolemia, most frequently in association with elevated TSH levels (Figure 1).

When stratified by thyroid status, patients with subclinical hypothyroidism had significantly higher cholesterol values compared to those with subclinical hyperthyroidism, whose lipid profiles tended to be comparatively lower. A weak, negative linear correlation was observed between TSH and FT4 levels, approaching but not reaching statistical significance (r = −0.221, *p* = 0.056) (Figure 2).

### 3.3. Thyroid Function and Stroke Severity

Stroke severity was assessed using the NIHSS score at admission. Serum TSH levels showed a significant, directly proportional correlation with the NIHSS score at admission in patients with acute ischemic stroke affecting the anterior circulation. Higher TSH values were associated with more severe neurological deficits at presentation (Figure 3).

In contrast, no statistically significant correlation was identified between TSH levels and NIHSS scores in patients presenting with lacunar infarction, posterior circulation stroke, or TIA. Patients diagnosed with TIA did not exhibit statistically significant correlations between serum levels of the studied biological markers and thyroid pathology (*p* = 0.826), FT4 (*p* = 0.418), or TSH (*p* = 0.345). This differential pattern suggests that thyroid dysfunction may exert greater influence in stroke subtypes driven by large-vessel atherosclerosis, whereas small-vessel or transient ischemic mechanisms appear less directly associated with thyroid status.

### 3.4. Carotid Atherosclerosis

Extracranial carotid Doppler ultrasonography revealed a statistically significant association between TSH levels and the severity of carotid stenosis. Serum TSH levels were able to discriminate between patients with no or minimal carotid atheromatosis (Grade 0) and those with moderate (Grade 1; 50–69% carotid stenosis) or severe carotid atheromatosis (Grade 2; ≥70% carotid stenosis), with statistical significance (*p* < 0.05) (Table 3). Importantly, the lowest measurable TSH value associated with moderate or severe stenosis was 4.7 mU/L, suggesting that vascular risk may increase even at modest elevations within the subclinical range (Figure 4).

To evaluate the association between subclinical thyroid dysfunction and the presence of moderate-to-severe carotid atheromatosis (Grade ≥ 1), a limited logistic regression analysis was conducted, adjusting only for patient gender. In this preliminary model, increasing serum TSH concentration was associated with significant carotid stenosis (Adjusted OR = 1.155; 95% CI: 1.050–1.271; *p* = 0.002). However, because this model lacks comprehensive adjustment for major clinical confounders—such as age, smoking status, hypertension, and diabetes mellitus—this result must be interpreted strictly as a preliminary association rather than identifying TSH as a definitive independent predictor. Similarly, while subclinical hyperthyroidism showed a statistical inverse association with advanced extracranial atherosclerosis (OR = 0.211; 95% CI: 0.043–0.996; *p* = 0.038), this finding must be interpreted with extreme caution due to the very small sample size of this subgroup (*n* = 13) and the high potential for residual confounding.

### 3.5. Atrial Fibrillation

Despite the lack of structural carotid disease, the majority of patients with subclinical hyperthyroidism (78%) presented with atrial fibrillation as an associated cardiovascular condition. All cases of atrial fibrillation were newly diagnosed at the time of hospital admission, and none of the patients were receiving anticoagulant therapy prior to admission. In contrast, atrial fibrillation was considerably less frequent among patients with subclinical hypothyroidism. This finding strongly suggests that ischemic events occurring in the hyperthyroid subgroup were predominantly cardioembolic in origin rather than atherosclerotic.

### 3.6. Functional Outcome at Discharge

Functional outcome at discharge was systematically evaluated using the Modified Rankin Scale (mRS). Patients with subclinical hypothyroidism demonstrated a significantly higher proportion of poor clinical outcomes (mRS 3–6) compared to the hyperthyroid subgroup, highlighting the negative prognostic impact of elevated TSH levels in the acute phase of ischemic stroke (Table 4, Figure 5).

### 3.7. Global Clinical Correlations

To provide a comprehensive overview of the interactions between endocrine, metabolic, and neurological parameters, a global Spearman rank correlation matrix was constructed for the entire study cohort. The resulting heatmap confirms the robust interdependencies between elevated TSH, hypercholesterolemia, atherosclerotic burden, and acute neurological severity.

Specifically, strong positive correlations were highlighted between serum TSH levels and Total Cholesterol, as well as between TSH and admission NIHSS scores. Total Cholesterol also demonstrated a moderate positive correlation with the severity of the neurological deficit. Conversely, FT4 levels exhibited weak and predominantly negative correlations with the other clinical variables, reinforcing the premise that TSH elevation is the primary biomarker associated with the observed metabolic and vascular changes in this cohort (Figure 6).

## 4. Discussion

The present study evaluated the association between subclinical thyroid dysfunction and cerebrovascular disease, focusing on lipid metabolism, carotid atherosclerosis, acute stroke severity, and short-term functional outcomes. Our findings suggest that even mild, subclinical alterations in thyroid function exert clinically relevant neurovascular effects and that the underlying pathophysiological mechanisms differ substantially according to the direction of the thyroid hormone imbalance [1,2]. Taken together, the findings support a robust dual-pathway model of cerebrovascular risk in subclinical thyroid dysfunction [3,4]. Subclinical hypothyroidism appears to actively promote atherosclerotic disease progression, impair acute neurovascular compensatory mechanisms, and increase stroke severity in large-vessel territories. Conversely, subclinical hyperthyroidism primarily predisposes to arrhythmia-mediated cardioembolic events without accelerating macroscopic atherogenesis.

A central finding of this study is the strong positive correlation observed between serum TSH and total cholesterol levels (r = 0.794, *p* < 0.001). This result aligns with and reinforces existing evidence demonstrating that subclinical hypothyroidism is intimately associated with dyslipidemia, particularly elevated total and low-density lipoprotein (LDL) cholesterol levels [5]. From a mechanistic standpoint, elevated TSH, even in the presence of normal FT4, directly downregulates the expression of hepatic LDL receptors and decreases the activity of cholesterol alpha-hydroxylase, thereby reducing cholesterol clearance [6,7]. Large meta-analyses, including those rigorously conducted by the Thyroid Studies Collaboration, have shown that increasing TSH concentrations within the subclinical range correlate with adverse lipid profiles and higher overall vascular risk [8]. Furthermore, pooled data from international cohorts have confirmed associations between subclinical thyroid dysfunction and multiple traditional risk factors, underscoring the systemic impact of early thyroid failure beyond isolated endocrine disruption [9,10,11,12].

Our observation that TSH levels correlated directly with the degree of extracranial carotid stenosis significantly expands upon these metabolic mechanisms, shifting the focus to structural vascular injury. Previous ultrasonographic studies have demonstrated increased carotid intima-media thickness (CIMT) and a higher prevalence of macroscopic carotid plaques in patients with subclinical hypothyroidism compared to euthyroid individuals [13,14]. Our findings of increased carotid atherosclerotic burden are strongly supported by recent literature: a comprehensive systematic review showed that subjects with subclinical hypothyroidism have significantly greater CIMT, indicating early atherogenic progression even in the absence of overt systemic thyroid disease [13]. Furthermore, a prospective controlled study demonstrated not only higher baseline CIMT in these individuals but also a measurable reduction in CIMT following levothyroxine therapy, suggesting that structural vascular changes may be partially reversible with hormonal normalization [15].

Furthermore, our logistic regression analysis bolsters this literature by identifying a strong association between elevated TSH and moderate-to-severe carotid stenosis (Adjusted OR = 1.155, *p* = 0.002). Although this preliminary model was adjusted only for gender and precludes defining TSH as a strictly independent predictor, the robust correlation suggests that elevated TSH actively contributes to structural atherogenesis. Recent experimental models explain this phenomenon by demonstrating the presence of functional TSH receptors on vascular endothelial and smooth muscle cells. Direct TSH binding induces endothelial dysfunction, reduces nitric oxide (NO) bioavailability, and promotes a pro-inflammatory state characterized by increased oxidative stress, key drivers of accelerated plaque formation and vulnerability [12,17].

From a strictly neurological perspective, another critical finding was the significant association between elevated TSH levels and acute stroke severity (assessed via admission NIHSS) in patients presenting with ischemic strokes localized in the anterior circulation. Large-vessel atherosclerosis represents the dominant pathophysiological mechanism in anterior circulation cerebral infarcts [18]. Therefore, the observed correlation between elevated TSH and higher NIHSS scores suggests that thyroid-mediated metabolic and endothelial disturbances contribute to more extensive vascular occlusion and, consequently, greater initial neurological deficits [19,20]. Furthermore, pre-existing chronic endothelial dysfunction in subclinical hypothyroidism may severely impair acute compensatory vasodilation and collateral network recruitment during the ischemic event, leaving the ischemic penumbra highly vulnerable to irreversible infarction. The absence of similar correlations in lacunar or posterior circulation strokes supports a mechanism-specific interpretation: small-vessel disease and posterior circulation events may be less dependent on macroscopic large-vessel atheromatosis and thus less directly influenced by TSH-driven lipid abnormalities. Additionally, the lack of significant correlations in patients with transient ischemic attacks (TIA) reflects the rapidly reversible nature of the ischemia in these cases, which does not result in established, permanent structural neurological deficits [21].

Expanding on the acute clinical presentation, our analysis of functional outcomes at discharge via the Modified Rankin Scale (mRS) revealed a substantially higher proportion of severe disability (mRS 3–6) in the subclinical hypothyroidism group. This highlights the negative prognostic value of elevated TSH in the acute and subacute phases of ischemic stroke [22]. Thyroid hormones play a pivotal role in central nervous system neuroplasticity, axonal regeneration, and remyelination following ischemic injury. Even subtle local deficiencies in thyroid hormone signaling at the tissue level, often masked by normal serum FT4 but heralded by elevated TSH, can impair neurogenesis and hinder functional neurological rehabilitation, providing a plausible biological explanation for the poorer discharge mRS scores observed in this cohort [23,24].

In contrast, subclinical hyperthyroidism demonstrated a fundamentally different neurovascular profile. While patients with suppressed TSH levels appeared to be protected against moderate or severe carotid stenosis (likely due to enhanced lipid clearance), they exhibited a strikingly high prevalence of newly diagnosed atrial fibrillation (78%) at stroke onset. Current guidelines from the European Thyroid Association (ETA) emphasize the strong link between low TSH levels and atrial electrical remodeling, a major independent risk factor for cardioembolic stroke [25]. Excess thyroid hormone activity, even at the subclinical level, enhances adrenergic sensitivity, increases atrial automaticity, and decreases the refractory period of atrial myocytes, strongly predisposing patients to paroxysmal or permanent arrhythmias and subsequent thromboembolism [26,27]. Therefore, while subclinical hyperthyroidism may attenuate large-vessel atherosclerotic progression, it simultaneously and significantly elevates the risk of severe cardioembolic cerebrovascular events [11].

These results have critical clinical implications for acute neurology and post-stroke care. Routine thyroid function testing in patients admitted to stroke units provides highly valuable information regarding underlying pathological mechanisms and risk stratification. The clinical implications of our findings are robustly supported by modern endocrinology guidelines regarding the management of subclinical hypothyroidism. Recent consensus statements, such as the European Thyroid Association (ETA) guidelines, strongly recommend the consideration of levothyroxine replacement therapy not only for TSH levels > 10 mU/L, but also for patients with subclinical hypothyroidism (TSH < 10 mU/L) when accompanied by high cardiovascular risk, age under 65, or significant dyslipidemia [28]. Given the strong correlation observed in our neurovascular cohort between elevated TSH, profound hypercholesterolemia, and increased carotid atherosclerotic burden, early therapeutic intervention with levothyroxine in stroke survivors may not only restore metabolic balance but could also play a critical role in secondary stroke prevention by mitigating long-term atherogenic progression and restoring endothelial reactivity [29]. Conversely, the detection of subclinical hyperthyroidism in a stroke unit must prompt rigorous, long-term cardiac rhythm surveillance (e.g., via Holter monitoring or implantable loop recorders) and rapid initiation of appropriate anticoagulation protocols [30,31].

## 5. Study Limitations

Several limitations must be acknowledged. First, the absence of an euthyroid control group substantially limits our ability to establish a definitive baseline for cardiovascular risk relative to the general population. Second, the total sample size is small (N = 80), and the subclinical hyperthyroidism subgroup is particularly limited (*n* = 13), making specific statistical estimates prone to instability. Third, the single-center, retrospective observational design restricts the ability to establish definitive causality. Fourth, grouping patients with transient ischemic attacks (TIA)—who inherently present with NIHSS and discharge mRS scores of 0—together with acute stroke patients may skew the general distributions in our severity and functional outcome analyses. Crucially, our logistic regression model evaluating carotid stenosis was adjusted only for gender; the lack of comprehensive adjustment for major cardiovascular confounders—specifically age, smoking status, hypertension, BMI, and diabetes—represents a significant methodological limitation [32,33]. Consequently, our findings highlight a robust preliminary association rather than confirming TSH as an independent predictor of atherogenesis. Furthermore, thyroid function was evaluated solely upon hospital admission. The severe physiological stress of an acute cerebral ischemic event can temporarily alter thyroid profiles, leading to non-thyroidal illness syndrome (euthyroid sick syndrome), which can occasionally mimic subclinical thyroid dysfunction [34,35]. Because we lacked pre-stroke baseline laboratory values, distinguishing true preexisting endocrine pathology from acute stress-induced neuroendocrine alterations remains a challenge. Finally, functional neurological outcomes were assessed only at discharge, lacking long-term longitudinal follow-up.

## 6. Conclusions

Subclinical thyroid dysfunction appears to be associated with cerebrovascular pathology through distinct mechanisms. Elevated TSH in subclinical hypothyroidism correlates with dyslipidemia and an increased carotid atherosclerotic burden, and is associated with greater initial stroke severity in large-vessel events. In contrast, subclinical hyperthyroidism is closely linked to newly diagnosed atrial fibrillation, pointing toward a predominantly cardioembolic mechanism of stroke. Given our methodological limitations, including the lack of comprehensive multivariable adjustments, these findings highlight subclinical thyroid dysfunction as a relevant neurovascular risk modifier that warrants further validation through large-scale, properly controlled prospective studies.

## Figures and Tables

**Figure 1:**
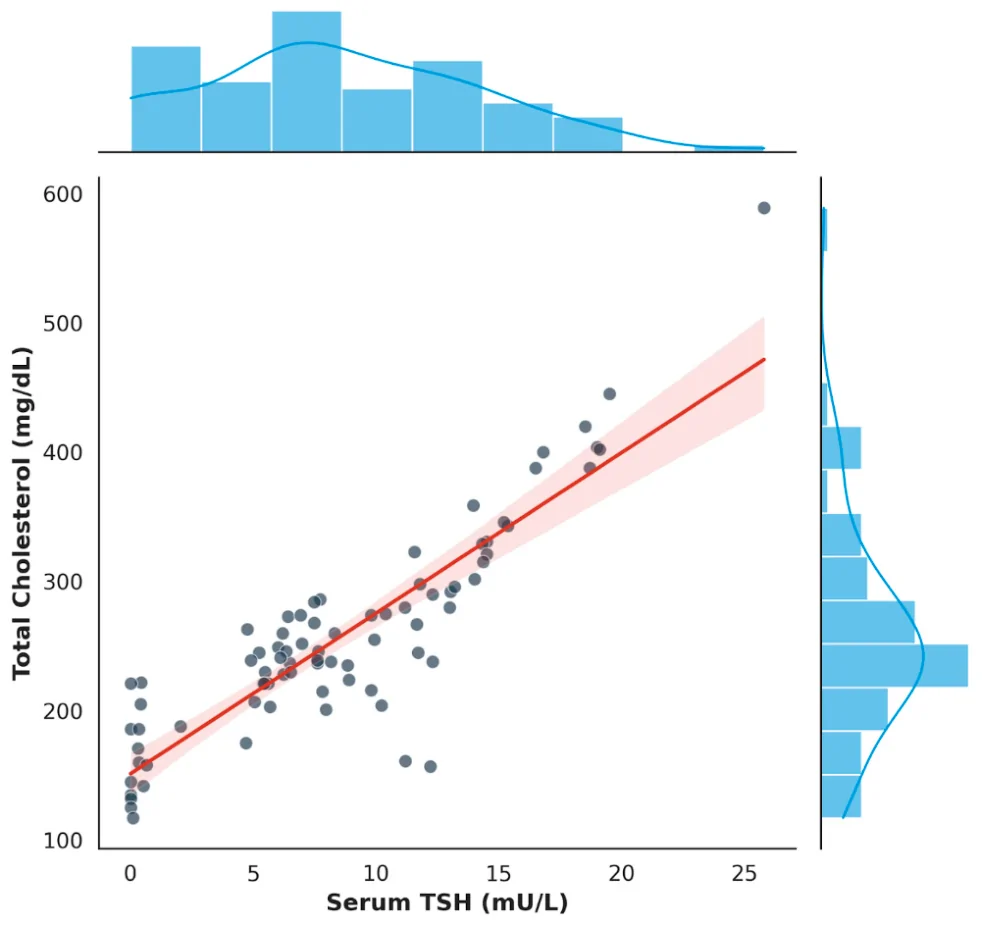
Linear correlation between serum TSH and Total Cholesterol.

**Figure 2:**
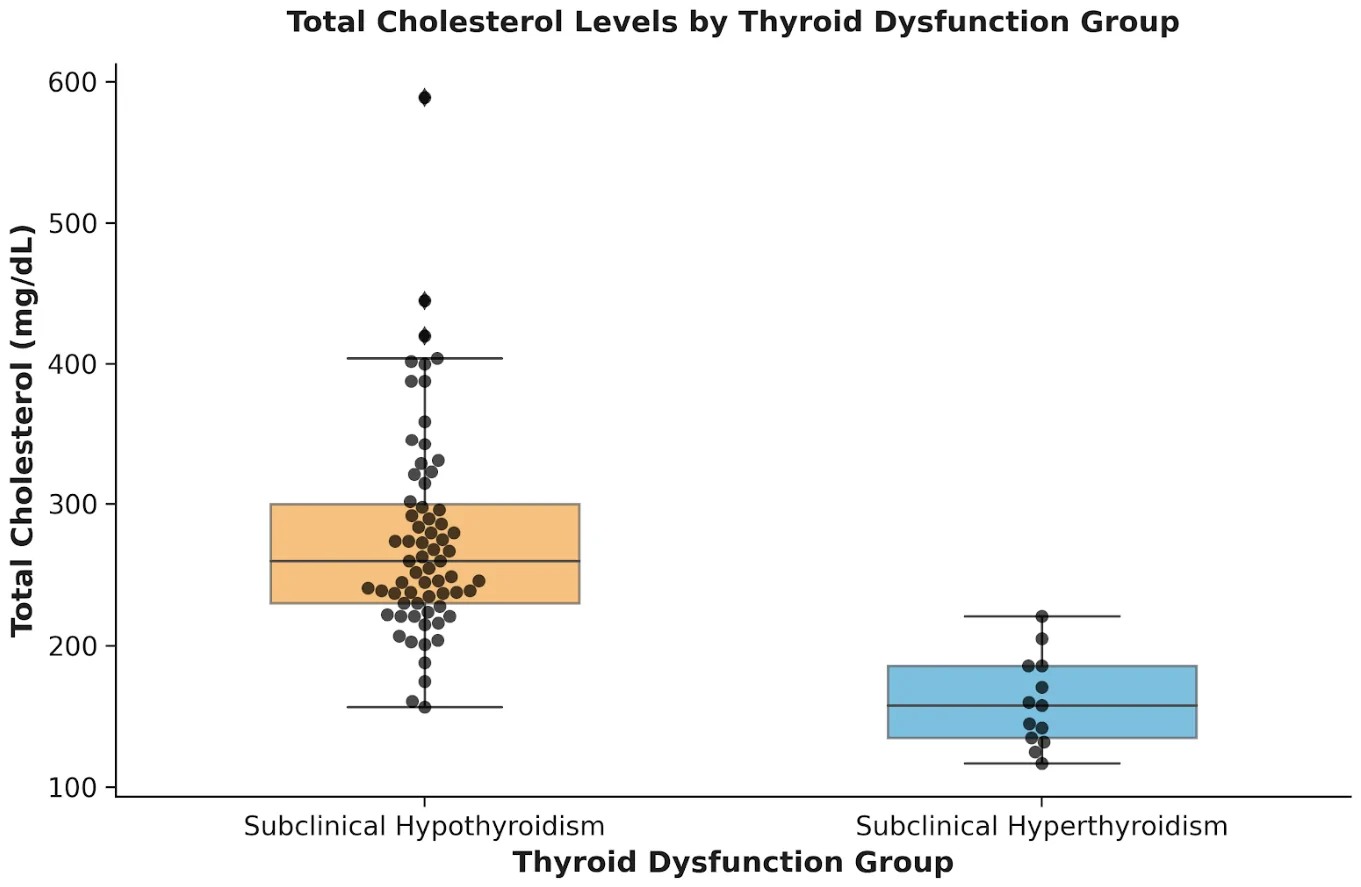
Distribution of Total Cholesterol levels stratified by thyroid dysfunction group.

**Figure 3:**
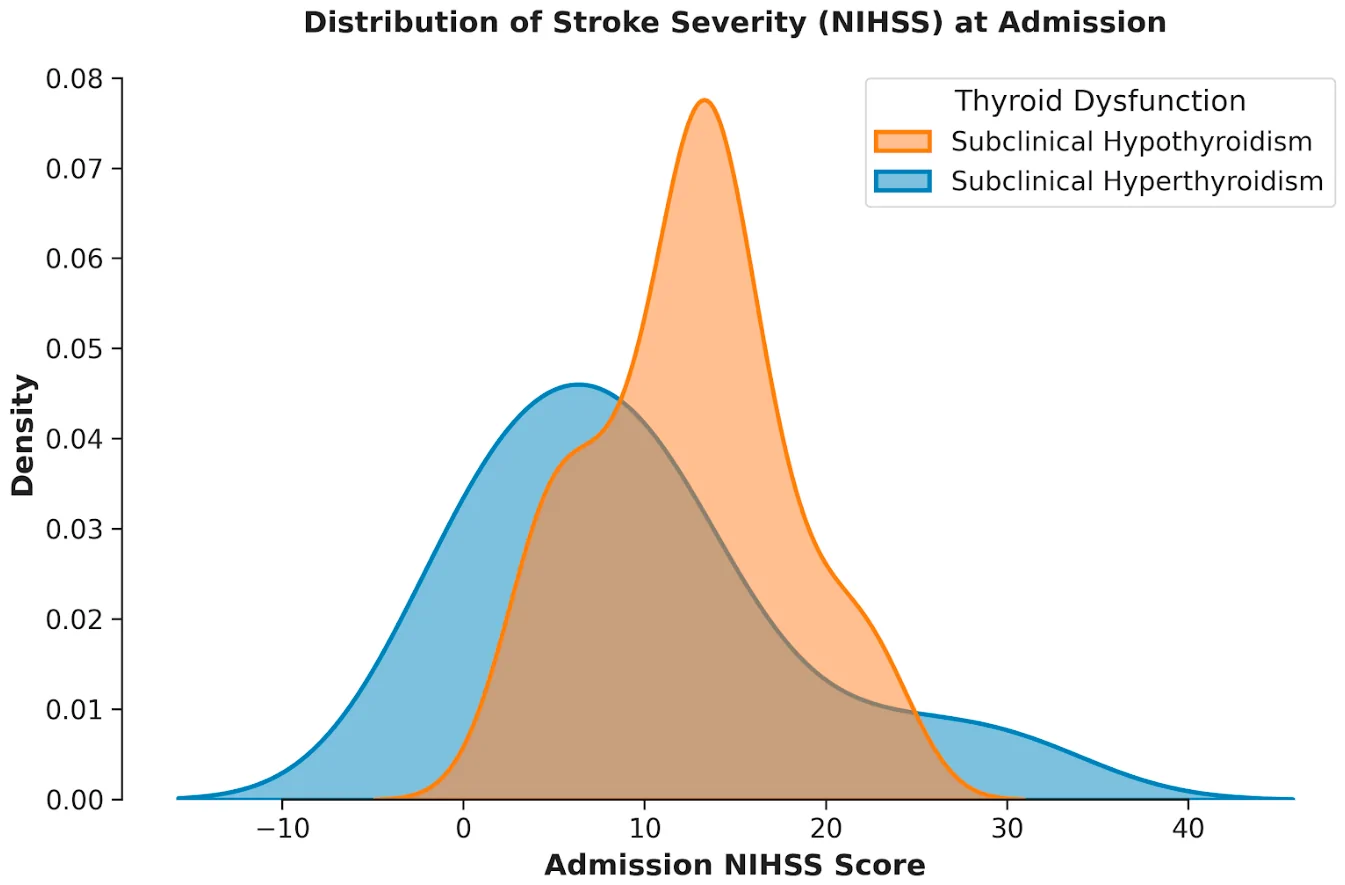
Distribution of admission stroke severity (NIHSS) stratified by thyroid status.

**Figure 4:**
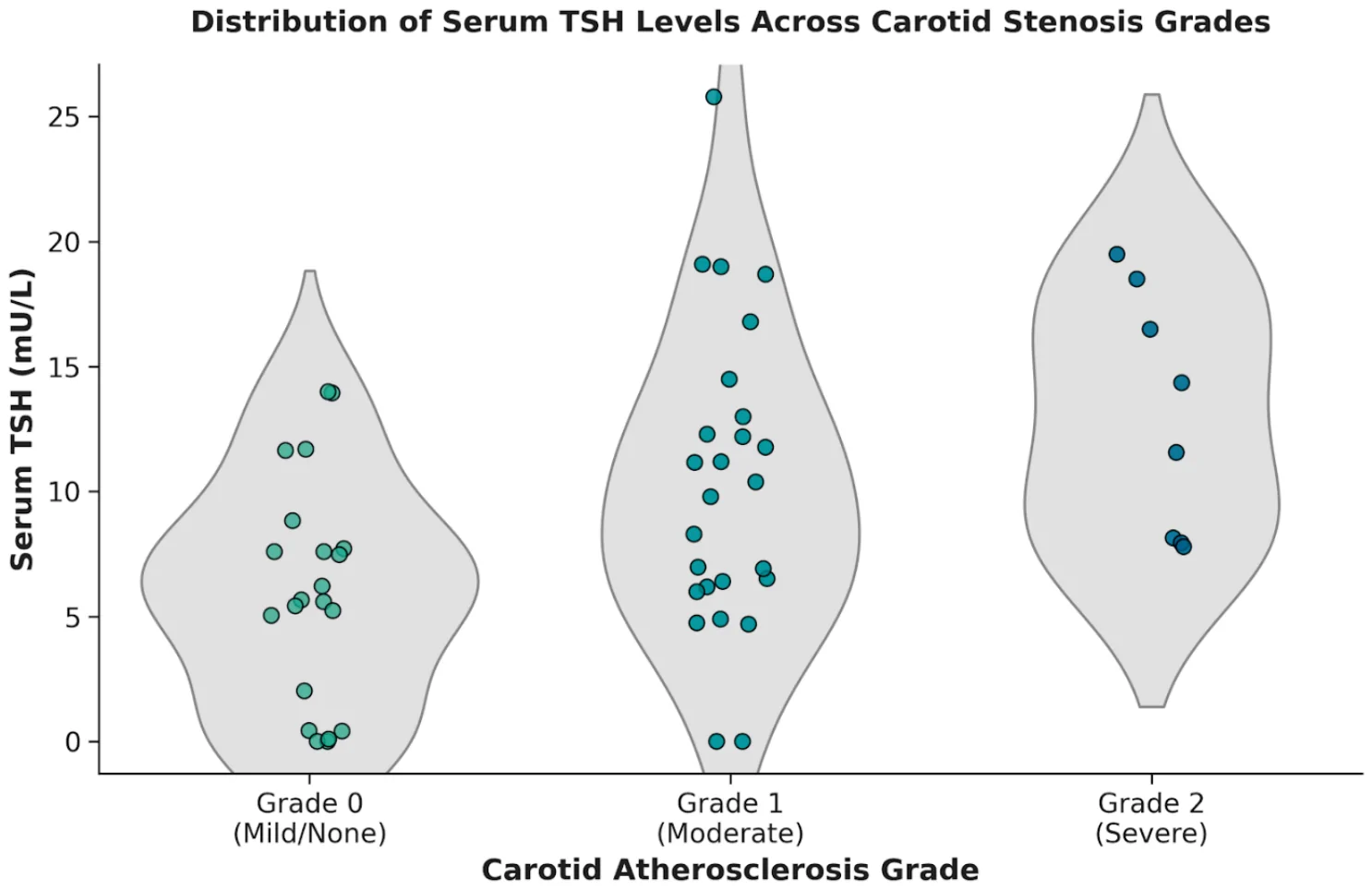
Distribution of serum TSH concentrations across carotid atherosclerosis grades.

**Figure 5:**
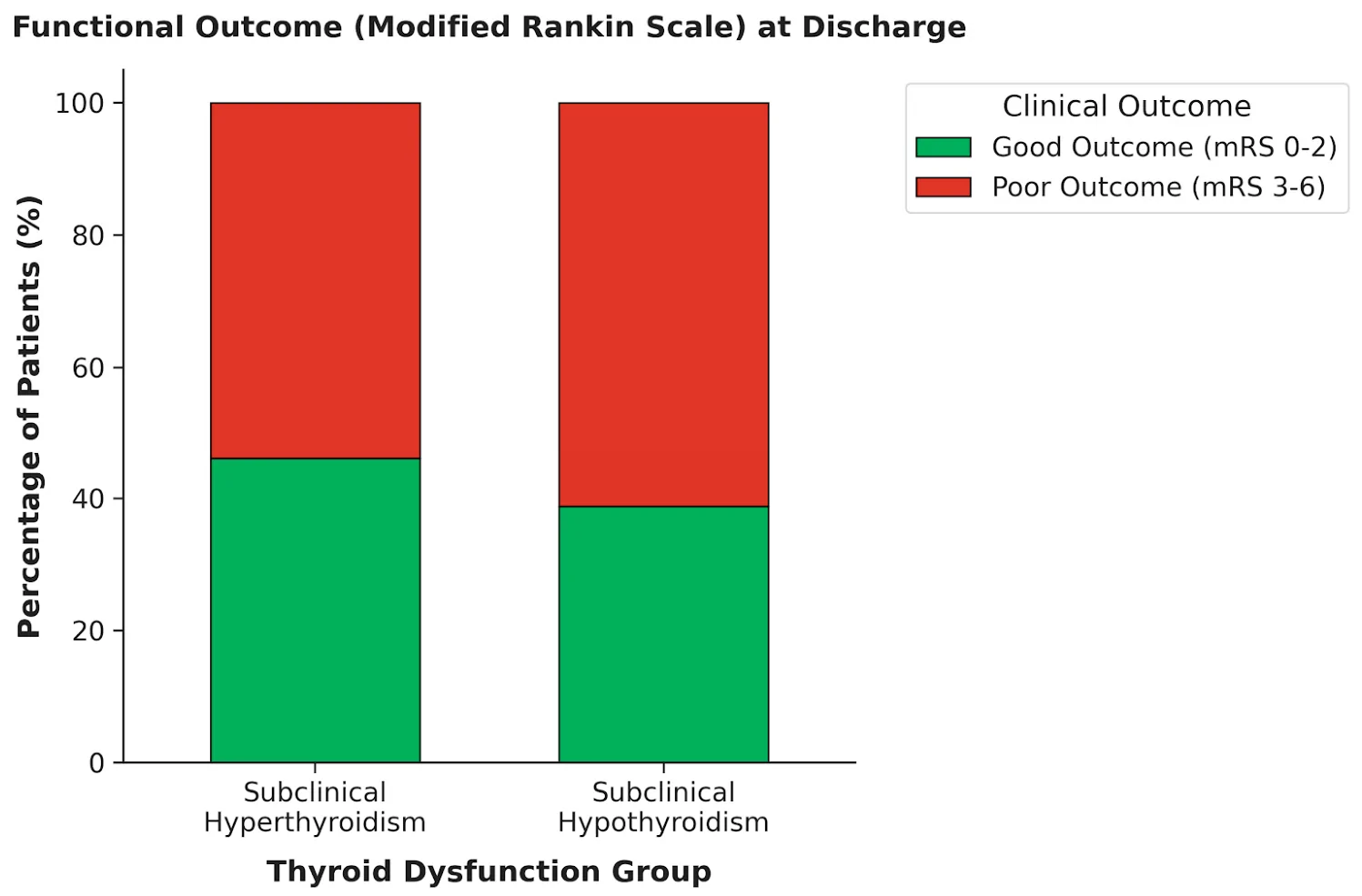
Functional outcome at discharge based on the Modified Rankin Scale (mRS).

**Figure 6:**
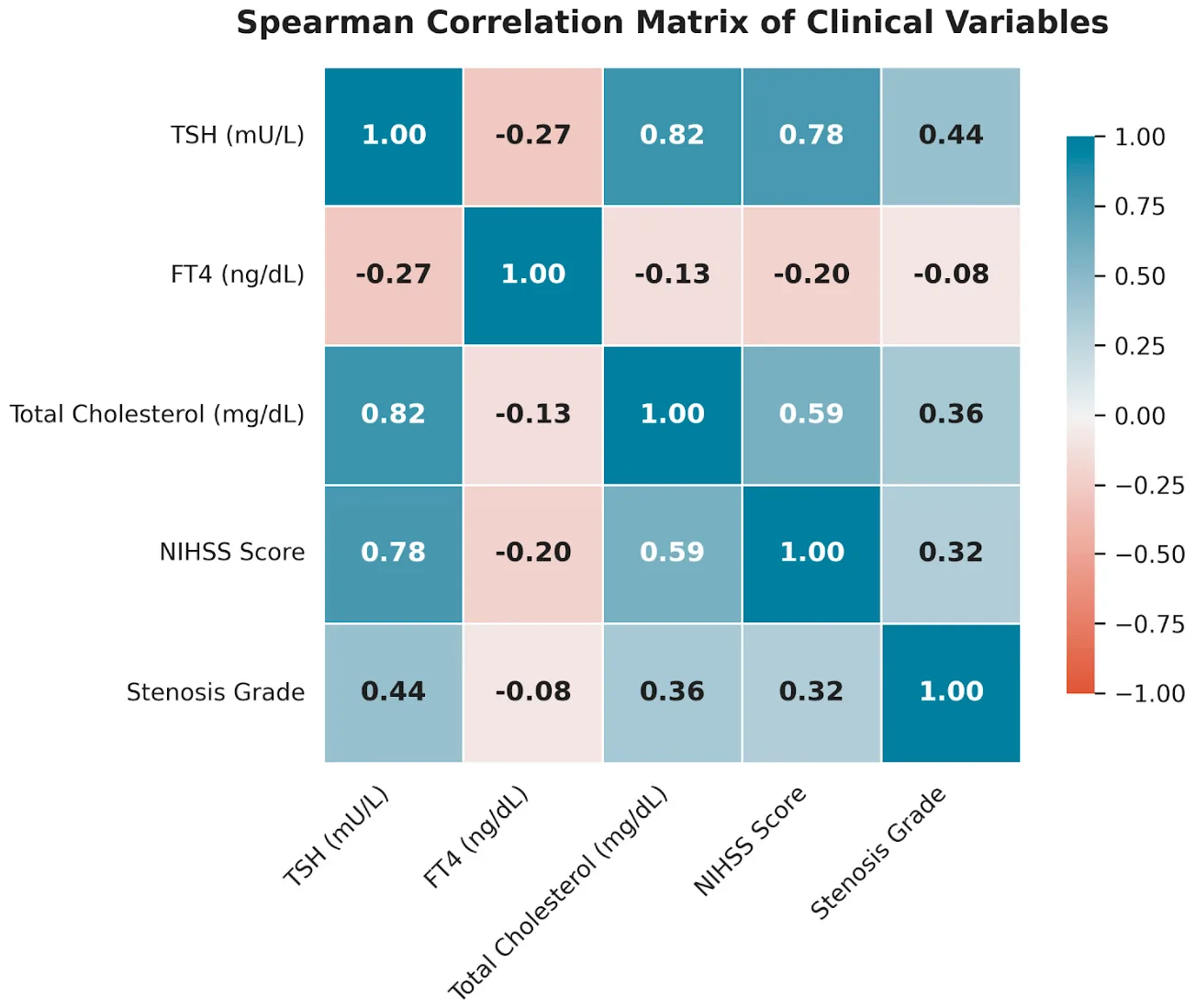
Spearman rank correlation matrix of clinical, metabolic, and neurological parameters.

**Table 1:** Baseline demographic and clinical characteristics of the study population.

Parameter	Subclinical Hyperthyroidism (*n* = 13)	Subclinical Hypothyroidism (*n* = 67)	*p*-Value
Gender (Female), *n* (%)	10 (76.9%)	57 (85.1%)	
Gender (Male), *n* (%)	3 (23.1%)	10 (14.9%)	
TSH (mU/L), mean ± SD	0.21 ± 0.23	10.14 ± 4.81	<0.001 *
FT4 (ng/dL), mean ± SD	1.54 ± 0.66	1.11 ± 0.32	0.003 *
Total Cholesterol (mg/dL), mean ± SD	160.23 ± 31.96	275.99 ± 74.13	<0.001 *
NIHSS at admission, median (IQR)	8.0 (3.0–12.0)	13.0 (7.5–16.0)	0.021 *

**Table 2:** Stroke Vascular Territory Distribution by Thyroid Dysfunction Group.

Parameter	Subclinical Hyperthyroidism	Subclinical Hypothyroidism
Total Patients (N)	13	67
Anterior Circulation, *n* (%)	7 (53.8%)	29 (43.3%)
Posterior/Other, *n* (%)	6 (46.2%)	38 (56.7%)

**Table 3:** Carotid ultrasound stenosis grade by thyroid dysfunction group.

Parameter	Subclinical Hyperthyroidism	Subclinical Hypothyroidism
Total Patients (N)	13	67
Grade 0 (Mild/None), *n* (%)	11 (84.6%)	35 (52.2%)
Grade 1 (Moderate), *n* (%)	2 (15.4%)	24 (35.8%)
Grade 2 (Severe), *n* (%)	0 (0.0%)	8 (11.9%)

**Table 4:** Functional outcome at discharge (Modified Rankin Scale).

Parameter	Subclinical Hyperthyroidism	Subclinical Hypothyroidism
Total Patients (N)	13	67
Good Outcome (mRS 0–2), *n* (%)	6 (46.2%)	26 (38.8%)
Poor Outcome (mRS 3–6), *n* (%)	7 (53.8%)	41 (61.2%)

## Data Availability

The data supporting this study are available upon request from the corresponding author. Due to privacy and ethical considerations, the data are not publicly accessible.
